# No Association between Antenatal Common Mental Disorders in Low-Obstetric Risk Women and Adverse Birth Outcomes in Their Offspring: Results from the CDS Study in Ghana and Côte D'Ivoire 

**DOI:** 10.1371/journal.pone.0080711

**Published:** 2013-11-18

**Authors:** Carola Bindt, Nan Guo, Marguerite Te Bonle, John Appiah-Poku, Rebecca Hinz, Dana Barthel, Stefanie Schoppen, Torsten Feldt, Claus Barkmann, Mathurin Koffi, Wibke Loag, Samuel Blay Nguah, Kirsten A. Eberhardt, Harry Tagbor, Eliezer N’Goran, Stephan Ehrhardt, for the International CDS Study Group

**Affiliations:** 1 Department of Child and Adolescent Psychiatry, University Medical Center Hamburg-Eppendorf, Hamburg, Germany; 2 Department of Epidemiology, Johns Hopkins Bloomberg School of Public Health, Baltimore, Maryland, United States of America; 3 Centre de Guidance Infantile, Institut National de Santé Publique, Abidjan, Côte d’Ivoire; 4 Department of Behavioural Sciences, School of Medical Sciences, Kwame Nkrumah University of Science and Technology, Kumasi, Ghana; 5 Clinical Research Unit, Bernhard Nocht Institute for Tropical Medicine, Hamburg, Germany; 6 Jean Lorougnon Guede University, Daloa, Côte d'Ivoire; 7 Infectious Disease Epidemiology, Bernhard Nocht Institute for Tropical Medicine, Hamburg, Germany; 8 Department of Child Health, Komfo Anokye Teaching Hospital, Kumasi, Ghana; 9 Department Community Health, School of Medical Sciences, Kwame Nkrumah University of Science and Technology, Kumasi, Ghana; 10 Research Unit of Parasitology and Parasite Ecology at Unité de Formation et de Recherche en Biosciences, Université de Cocody, Abidjan, Côte d’Ivoire; The University of Queensland, Australia

## Abstract

**Background:**

Evidence linking common mental disorders (CMD) in pregnant women to adverse birth outcomes is inconsistent, and studies often failed to control for pregnancy complications. This study aimed to explore the association between antenatal depression and anxiety symptoms and birth outcomes in a low-obstetric risk sample of mother/child dyads in Ghana and Côte d’Ivoire.

**Methods:**

In 2010-2011, a prospective cohort of 1030 women in their third trimester in Ghana and Côte d’Ivoire was enrolled. Depression and anxiety were assessed in the third trimester using the Patient Health Questionnaire depression module and the 7-item Generalized Anxiety Disorder scale. 719 mother/child dyads were included in the analysis. We constructed multivariate regression models to estimate the association between CMD and low birth weight (LBW), and preterm birth (PTB) to control for potential confounders.

**Results:**

The prevalence of depression and anxiety symptoms were 28.9% and 14.2% respectively. The mean birth weight was 3172.1g (SD 440.6) and the prevalence of LBW was 1.7%. The mean gestational age was 39.6 weeks and the proportion of PTB was 4%. Multivariate linear regression revealed no significant association between maternal depression (B=52.2, 95% CI -18.2 122.6, p=0.15) or anxiety (B=17.1, 95% CI -74.6 108.7, p=0.72) and birth weight. Yet, low socio-economic status, female sex of the child, and younger maternal age were associated with lower birth weight. Multivariate logistic regression suggested no significant association between maternal depression (OR: 2.1, 95% CI 0.8 5.6, p=0.15) or anxiety (OR: 1.8, 95% CI 0.6 5.5, p=0.29) with PTB.

**Conclusions:**

Our data suggests that depression and/or anxiety in the 3^rd^ trimester of pregnancy are not independent predictors of adverse birth outcomes in low obstetric risk women. The role of pregnancy complications as confounders or effect modifiers in studies of maternal CMD and their impact on birth outcomes should be investigated.

## Introduction

Adverse birth outcomes such as low birth weight, preterm birth and low Apgar scores are important predictors of infant survival and of long term health, including neurodevelopmental, emotional, and cognitive functioning and educational outcome [1-3]. 

Adverse birth outcomes have been linked to common mental disorders (CMD) in pregnant women. On average at least one in six pregnant women will experience CMD, especially depression and anxiety [4-6] in antenatal clinics worldwide. Maternal depression and anxiety were found to result in poor self-care and unhealthy behaviors [7], abnormal stress levels and hormonal alterations [8], and impaired immune function [9], all of which may directly or indirectly lead to poor birth outcomes. Important adverse birth outcomes are low birth weight (LBW), preterm birth (PTB), and low Apgar score. 

Evidence linking maternal CMD symptoms to adverse birth outcomes is inconsistent and inconclusive. Studies in low-income countries, mainly from South-East Asia, found larger effects of maternal mental health on adverse birth outcomes than studies in high-income countries [10-15]. In high-income countries, depressed women appeared to have a higher risk of PTB than non-depressed women only if their socio-economic status (SES) was low [10]. In sub-Saharan Africa where stressors due to poverty and maternal ill-health prevail, CMD during pregnancy may be associated with particularly poor perinatal outcomes. Yet, the only study identified from this region did not confirm this assumption in Ethiopian women [16]. Overall, the comparability of results is complicated by different study designs, verification of depression and anxiety, timing of assessment and adjustment for confounders like SES.

The aim of this study was to explore the association between women’s depression and anxiety symptoms during the third trimester of pregnancy and birth outcomes in a sample of African mother/child dyads from the Child Development Study (CDS) in Ghana and Côte d’Ivoire [17]. We recently reported a prevalence of antepartum depression of 27% and 33%, and anxiety symptoms of 11% and 17% in this sample, respectively [17]. Antepartum and postpartum depression was associated with the febrile illness in their offspring [18]. To evaluate the unique contribution of maternal depression and anxiety on birth outcomes, maternal risk factors known to be associated with impaired pregnancy course and sequelae for the neonate were eliminated by restriction to a low-obstetric risk sample [19,20]. We hypothesized to find more unfavorable birth outcomes in children born to mothers who scored high on the general depression and anxiety symptom scales during late pregnancy.

## Methods

### Study design and setting

CDS is a longitudinal birth cohort of mothers and children in Ghana and Côte d’Ivoire. The primary aim of the study is to investigate the effect of maternal infectious and non-infectious disease on child development. Details of the study design have been described elsewhere [17]. In brief, women in their last trimester of pregnancy were consecutively recruited in two large hospitals, the Komfo Anokye Teaching Hospital in Kumasi, Ghana, and the Abobo Community Hospital in Abidjan, Côte d’Ivoire during antenatal care visits between March 2010 and December 2011. While the Komfo Anokye Teaching Hospital serves a mixed population in Ghana’s second largest city, the Abobo Community Hospital provides healthcare to an underprivileged population affected by civil war. After birth, the children of included mothers were enrolled into a prospective, longitudinal birth cohort for a two-year follow-up period. Here, we report data on the association between depression and anxiety status during the third trimester of pregnancy and PTB, LBW, and low Apgar scores in newborn children to these mothers.

### Ethics statement

 This study was conducted in accordance with the ethical principles of the Declaration of Helsinki. It was approved by the ethical committee of the Kwame Nkrumah University of Science and Technology in Kumasi, Ghana, the National Ethical Committee in Côte D’Ivoire, and the respective committee of the Chamber of Physicians in Hamburg, Germany. Generally, persons suffering from non-psychotic depression are able to understand and consent to study requirements. All participating mothers gave written informed consent.

### Participants and procedures

**Figure 1:**
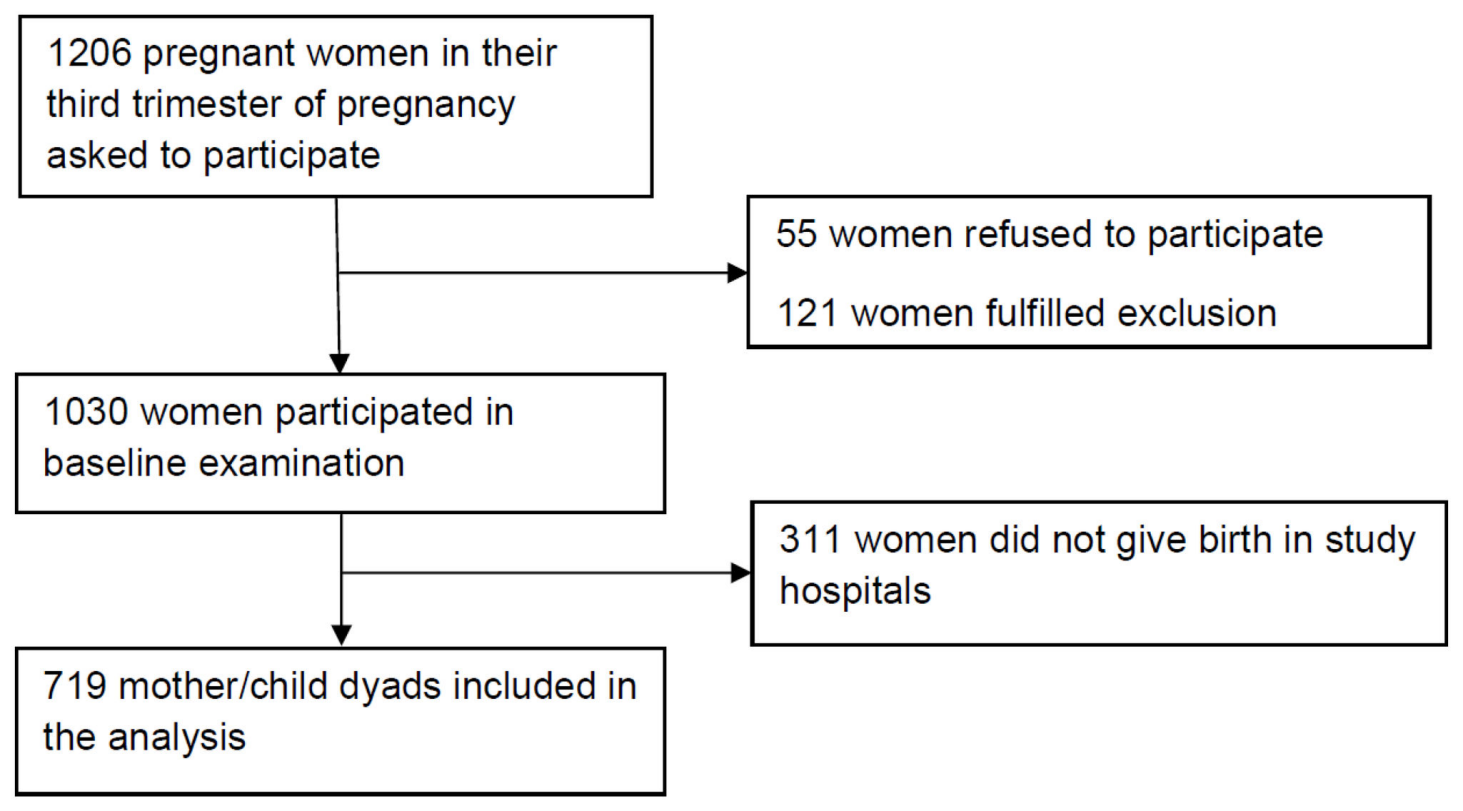
Study flow.

All women in their third trimester of pregnancy, based on gestational age assessed at the antenatal clinic, residing within a distance of ≤ 5 km around the hospitals were eligible for participation. Women under 18 years of age, with multiple pregnancies, chronic physical disease, or other risk factors like severe complications during pregnancy due to diabetes, hemorrhage, hypertension, and preeclampsia were excluded from the study (restriction to a low-obstetric risk cohort) [20]. The study flow is displayed in Figure 1. Due to political instability and resumed fighting in Côte d’Ivoire during the study period, 311 mothers did not give birth in the designated hospitals and could therefore not be followed-up. The missing mothers were, on average, more depressed, more anxious, younger, and of lower SES. 719 singletons and live births were included in the analysis. Women who scored high in the PHQ-9 and the GAD-7 were referred to local mental health professionals for further assessment and consultation. No psychotropic medications were prescribed.

### Measurements

#### Assessment of birth outcomes

Children’s weight was measured at birth. LBW was defined as a birth weight of less than 2500 g. Gestational age was estimated according to second trimester ultrasound screening as documented in the hospital charts. PTB was defined as gestational age less than 37 0/7 weeks. The Apgar score was assessed by a trained midwife 5 minutes after birth. A low Apgar score was defined as less than 7 at 5 minutes.

#### Assessment of antepartum depression and anxiety symptoms

The explanatory variables were antepartum maternal depression and anxiety. Depression was screened for with the Patient Health Questionnaire depression module (PHQ-9) [21]. The PHQ-9 refers to the past two weeks and assesses the presence and severity of the nine DSM-IV depression criteria, comprising emotional, cognitive, and functional somatic symptoms [22]. Response options generate a continuous raw score ranging from 0 (no symptoms) to 27 (all symptoms present nearly every day); with scores 5-9 representing mild, 10–14 moderate and 15–27 moderately severe to severe depression symptoms. The PHQ-9 has been validated for use in primary care and in the general population in both high-income and low-income settings. In rural postpartum Ghanaian women, the PHQ-9 proved superior to other common depression screening measures against a semi-structured clinical interview [23] as reference standard. A threshold score of ≥ 10 had a sensitivity of 88% and a specificity of 88% for major depression [24] and was used for case classification. The reliability of the raw score was Cronbachs α = .69 for the Ghanaian women and .64 for the women from Côte d’Ivoire. Anxiety was assessed using the GAD-7, a screening questionnaire for generalized anxiety disorder (GAD) according to seven DSM-IV symptoms [25]. The GAD-7 has a response set similar to the PHQ-9, comprising emotional and cognitive symptoms of anxiety during the past two weeks. Item scores range from 0 to 21, with 5 - 9 representing mild, 10 - 14 moderate, and 15 - 21 severe levels of anxiety. The threshold score of ≥10 had a sensitivity of 89% and a specificity of 82% for a generalized anxiety disorder and was used for case classification. The GAD-7 has been validated in western primary care, but not in antepartum populations or in African settings, as research in antepartum anxiety is still in its early stages [26,27]. In our study, the reliability of the raw score was Cronbachs α = .68 in both countries. Both depression assessed by the PHQ-9 and anxiety measured by the GAD-7 do not refer to a clinical diagnosis but to the result of a robust screening procedure in an epidemiological study with the above-mentioned properties. 

#### Assessment of socioeconomic, anthropometric, and obstetric characteristics of the women

 Other variables known to be associated with birth outcomes including SES, infant sex, previous pregnancy complications, maternal height, age, infections, hemoglobin level, malaria treatment, and caesarian section were controlled for. Maternal smoking and drinking status were not collected because both risk factors are known to be very rare among women in the two countries [28,29]. Marital status was not included into our analyses because the study population was homogenously married. An SES score was constructed using a principal component analysis based on ownership of refrigerator, car, and bed net; toilet type, maternal education, and spouse education. Women’s hemoglobin level and height were measured using standard techniques. 

### Statistical analysis

 We first assessed correlations between birth weight, gestational age, Apgar score at 5 minutes, PHQ-9 continuous raw score, and GAD-7 continuous raw score. Children’s average birth weight, gestational age and Apgar score were then compared between depressed (PHQ-9 ≥10) vs. non-depressed (PHQ-9 <10) mothers and anxious (GAD-7 ≥10) vs. non-anxious (GAD-7 <10) mothers using ANOVA. Univariate linear regressions were conducted to identify potential predictors associated with children’s birth weight. Multivariate linear regression models were conducted to control the simultaneous confounding effects of possible predictors. Model 1 shows the role of depressive symptoms (PHQ-9 ≥10) and Model 2 shows anxiety symptoms (GAD-7 ≥10) as predictors of children’s birth weight. The same analytic strategy was followed to assess predictors of PTB using logistic regression.

## Results

 The prevalence of depression and anxiety symptoms were 28.9% and 14.2% respectively among all women in the third trimester of pregnancy. Anxiety was more common at higher ages and in mothers from Côte d’Ivoire. The depressed mothers weighed more and had a larger BMI than the non-depressed mothers. The depressed and anxious mothers did not differ from healthy mothers in SES distribution, hemoglobin level, height, number of complications during previous pregnancies and caesarian section delivery (Table 1).

**Table 1:** Baseline characteristics of the study mothers by antepartum depression and anxiety status.

**Variable**	**Non- depressed n = 510**	**Depressed n = 207**	**p**	**Non-anxious n = 614**	**Anxious n = 102**	**p**	**Total n = 717**
**Socioeconomic**							
Age(m± SD)	28.8 ± 5.3	29.7 ± 5.9	0.06	28.8 ± 5.4	30.6 ± 5.9	0.00	29.1 ± 5.5
Country (%)			0.22			0.04	
Ghana	218 (42.8)	79 (37.8)		264 (42.9)	33 (32.0)		297 (41.3)
Côte d’Ivoire	292 (57.3)	130 (62.2)		351 (57.1)	70 (68.0)		422 (58.7)
SES (%)			0.12			0.06	
Top 20%	155 (31.4)	51 (25.1)		186 (31.4)	20 (19.6)		206 (29.6)
Middle 50%	198 (40.2)	80 (39.4)		232 (39.1)	46 (45.1)		278 (39.9)
Bottom 30%	140 (28.4)	72 (35.5)		175 (29.5)	36 (35.3)		212 (30.5)
**Anthropometric and obstetric**							
Hemoglobin ± SD (g/dL)	11.1 ± 1.2	10.9 ± 1.2	0.13	11.1 ± 1.1	10.8 ± 1.3	0.08	11.0 ± 1.2
Weight ± SD (kg)	70.1 ± 11.4	73.3 ± 13.9	0.00	70.9 ± 12.0	72.0 ± 13.7	0.38	71.0 ± 12.3
Height ± SD (cm)	161.0 ± 6.0	161.5 ± 6.5	0.27	161.1 ± 6.1	161.5 ± 6.2	0.52	161.1 ± 6.1
BMI ± SD	27.0 ± 4.3	28.1 ± 5.1	0.01	27.3 ± 4.5	27.5 ± 4.7	0.67	27.4 ± 4.6
Previous pregnancy complications (%)	113 (23.3)	53 (26.1)	0.43	138 (23.5)	28 (28.0)	0.33	166 (24.2)
Caesarian section(%)	94 (18.5)	37 (17.9)	0.84	111 (18.1)	20 (19.6)	0.72	131 (18.4)
PHQ-9 mean score ± SD	5.2 ± 2.5	13.4 ± 2.9	<0.001	6.7 ± 4.1	12.4 ± 4.3	<0.001	7.6 ± 4.6
GAD-7 mean score ± SD	4.1 ± 3.1	7.9 ± 4.3	<0.001	4.0 ± 2.6	12.2 ± 2.4	<0.001	5.2 ± 3.9

Notes. Depressed, PHQ-9 ≥ 10; Anxious, GAD-7 score ≥ 10; PHQ-9, Patient Health Questionnaire depression module; GAD, generalized anxiety disorder; SES, socio-economic status; BMI, body mass index

Table 2 shows the correlation coefficients between birth weight, gestational age, Apgar score at 5 minutes, PHQ-9 continuous raw score, and GAD-7 continuous raw score. The Apgar score was weakly correlated with depression and anxiety while birth weight and gestational age were not correlated with maternal mental health status. Table 3 shows the birth outcomes by maternal antepartum depression and anxiety status. The mean birth weight was 3172.1g (SD 440.6) and prevalence of LBW was 1.7%. The mean gestational age was 39.6 weeks and the proportion of PTB was 4%. We did not find any significant difference between depressed and non-depressed, and anxious and non-anxious women in terms of the average birth weight and head circumference of their children and gestational age at delivery. The average Apgar score was 8.5 in the children of anxious mothers and 8.7 in the children of non-anxious mothers (p=0.025), but the difference was not clinically meaningful. 

**Table 2:** Pearson Correlation coefficients.

	Birth Weight	Gestational Age	Apgar score	PHQ-9 score	GAD-7 score
Birth weight	1				
Gestational age	0.2481*	1			
Apgar score	-0.0380	-0.0414	1		
PHQ-9 score	0.0445	-0.0326	-0.1059*	1	
GAD-7 score	0.0182	-0.0309	-0.1019*	0.5420*	1

* p<0.05

**Table 3:** Comparison of birth outcomes by maternal antepartum depression and anxiety status.

**Outcome**	**Non- depressed n = 510**	**Depressed n = 207**	**p**	**Non-anxious n = 614**	**Anxious n = 102**	**p**	**Total n = 717**
Birth weight (g, mean ± SD)	3158.2 ± 434.8	3206.5 ± 453.9	0.18	3168.8 ± 443.5	3191.2 ± 426.5	0.64	3171.9 ± 440.9
< 2500 g (%)	9 (1.8)	3 (1.5)	0.22	12 (2.0)	0 (0.0)	0.45	12 (1.7)
2500-2999 g (%)	163 (32.0)	52 (25.1)		186 (30.3)	29 (28.4)		215 (30.0)
3000-3499 g (%)	207 (40.6)	100 (48.3)		257 (41.9)	49 (48.0)		306 (42.7)
>= 3500 g (%)	131 (25.7)	52 (25.1)		159 (25.9)	24 (23.5)		183 (25.6)
Gestational age at delivery (weeks, mean ± SD)	39.7 ± 1.8	39.6 ± 1.9	0.56	39.6 ± 1.9	39.5 ± 2.1	0.70	39.6 ± 1.9
< 34 wks (%)	4 (1.1)	0 (0.0)	0.21	4 (0.9)	0 (0)	0.25	4 (0.8)
34-36 wks (%)	9 (2.5)	8 (5.4)		12 (2.8)	5 (6.5)		17 (3.4)
37-38 wks (%)	72 (20.2)	33 (22.5)		85 (20.0)	20 (26.0)		105 (20.9)
38-39 wks (%)	65 (18.3)	31 (21.1)		85 (20.0)	11 (14.3)		96 (19.1)
> 39 wks (%)	206 (57.9)	75 (51.0)		240 (56.3)	41 (53.3)		281 (55.9)
Head circumference (cm, mean ± SD)	33.3 ± 1.8	33.3 ± 1.6	0.77	33.4 ± 1.8	33.2 ± 1.5	0.48	33.3 ± 1.7
Apgar score at 5 minutes (mean ± SD)	8.7 ± 0.6	8.6 ± 0.7	0.08	8.7 ± 0.6	8.5 ± 0.8	0.03	8.7 ± 0.6
< 7 (%)	0 (0.0)	2 (1.0)	0.11	0	2 (2.0)	0.03	2 (0.3)
7-9 (%)	490 (96.1)	199 (96.1)		593 (96.4)	95 (94.1)		689 (96.1)
10 (%)	20 (3.9)	6 (2.9)		22 (3.6)	4 (4.0)		26 (3.6)

Notes. Depressed, PHQ-9 ≥ 10; Anxious, GAD-7 score ≥ 10

 Univariate linear regression revealed no significant association between maternal depression or anxiety and children’s birth weight (Table 4). Being Ghanaian, belonging to the top 30% with respect to SES, increased maternal age, and male gender of the child were significantly associated with higher birth weight. In the multivariate analyses, neither maternal depression nor anxiety predicted children’s birth weight adjusted for country, SES, maternal age and child gender. Boys were on average 147g heavier at birth than girls when country, SES, maternal age, and maternal mental disorders were held constant. One year increase in maternal age was associated with 7g increase in children’s birth weight controlling for other factors. Children whose mothers belong to the top 30% SES group were 200g heavier at birth than children whose mothers belong to the lowest 30% SES group, adjusting for other factors.

**Table 4:** Univariate and multivariate linear regression for birth weight.

	Univariate			Multivariate^1^			Multivariate^2^		
	B	95% CI	*P*	B	95% CI	*P*	B	95% CI	*P*
Depression (Ref: Non-depressed)	48.4	-22.9, 119.6	0.18	52.2	-18.2, 122.6	0.15	-	-	-
Anxiety (Ref: Non-anxious)	22.4	-70.2, 115.0	0.64	-	-	-	17.1	-74.6, 108.7	0.72
Country (Ref: Côte d’Ivoire)	105.1	39.9, 170.2	0.00	-5.6	-89.0, 77.9	0.90	-5.2	-88.9, 78.6	0.90
SES (Ref: lowest 30%)									
Middle 40%	-2.2	-79.7, 75.3	0.96	15.8	-69.9, 95.6	0.70	14.1	-65.8, 94.1	0.73
Top 30%	177.8	94.7, 260.9	<0.001	202.2	96.7, 307.7	<0.001	199.4	93.7, 305.1	<0.001
Child sex (Ref: Female)	144.7	80.8, 208.5	<0.001	146.6	82.6, 210.5	<0.001	147.6	83.5, 211.6	<0.001
Maternal age	8.4	2.6, 14.3	0.01	7.1	1.3, 13.0	0.02	7.3	1.4, 13.2	0.02
Maternal hemoglobin	-1.6	-36.4, 33.2	0.93	-	-	-	-	-	-
Previous pregnancy complications	47.5	-29.6, 124.6	0.23	-	-	-	-	-	-
Maternal IPTp use	14.5	-56.4, 85.4	0.69	-	-	-	-	-	-
Maternal malaria treatment	-24.0	-95.9, 47.8	0.51	-	-	-	-	-	-
Maternal diarrhea	-44.4	-122.8, 34.0	0.27	-	-	-	-	-	-
Maternal fever	25.2	-42.7, 93.1	0.47	-	-	-	-	-	-
Maternal urinary tract infection	-23.1	-108.8, 62.5	0.60	-	-	-	-	-	-
Maternal vaginal infection	-24.7	-92.6, 43.3	0.48	-	-	-	-	-	-
Maternal HIV infection	-149.3	-369.5, 70.8	0.18	-	-	-	-	-	-
Maternal height	5.1	-0.2, 10.4	0.06	-	-	-	-	-	-
Delivery by caesarian section	82.9	-0.9, 166.8	0.05	-	-	-	-	-	-

Notes: Depression, PHQ-9 score ≥ 10; anxiety, GAD-7 score ≥ 10; SES, socio-economic status; IPTp, intermittent preventive treatment in pregnancy

1 Multivariate linear regression using depression as predictor

2 Multivariate linear regression using anxiety as predictor

 Univariate logistic regression revealed no significant association between maternal depression or anxiety and PTB (Table 5). Being Ivorian, lower SES and antepartum maternal vaginal infection were associated with increased likelihood for PTB. In the multivariate analyses, neither maternal depression nor anxiety was significantly associated with children’s PTB adjusted for country, SES, and maternal vaginal infection.

**Table 5:** Univariate and multivariate logistic regression for PTB.

	Univariate			Multivariate^1^			Multivariate^2^		
	OR	95% CI	*P*	OR	95% CI	*P*	OR	95% CI	*P*
Depression (Ref: Non-depressed)	1.6	0.7, 4.1	0.30	2.1	0.8, 5.6	0.15	-	-	-
Anxiety (Ref: Non-anxious)	1.9	0.7, 5.2	0.25	-	-	-	1.8	0.6, 5.5	0.29
Country (Ref: Côte d’Ivoire)	0.3	0.1, 0.7	0.01	0.6	0.2, 2.3	0.47	0.6	0.2, 2.5	0.52
SES (Ref: lowest 30%)									
Middle 40%	0.5	0.2, 1.3	0.16	0.5	0.2, 1.6	0.25	0.5	0.2, 1.5	0.22
Top 30%	0.2	0.1, 0.8	0.03	0.3	0.1, 1.8	0.21	0.3	0.1, 1.8	0.20
Child sex (Ref: Female)	0.7	0.3, 1.8	0.46	-	-	-	-	-	-
Maternal age	1.0	0.9, 1.1	0.67	-	-	-	-	-	-
Maternal hemoglobin	1.1	0.6, 2.1	0.75	-	-	-	-	-	-
Previous pregnancy complications	0.3	0.1, 1.4	0.12	-	-	-	-	-	-
Maternal IPTp use	1.1	0.4, 3.1	0.83	-	-	-	-	-	-
Maternal malaria treatment	1.1	0.4, 2.9	0.87	-	-	-	-	-	-
Maternal diarrhea	0.7	0.2, 2.6	0.64	-	-	-	-	-	-
Maternal fever	1.8	0.7, 4.5	0.21	-	-	-	-	-	-
Maternal urinary tract infection	1.1	0.3, 3.9	0.87	-	-	-	-	-	-
Maternal vaginal infection	3.0	1.2, 7.3	0.02	2.4	0.9, 6.7	0.10	2.3	0.8, 6.4	0.11
Maternal HIV infection	5.1	0.6, 46.1	0.15	-	-	-	-	-	-
Maternal height	1.0	0.9, 1.1	0.72	-	-	-	-	-	-
Delivery by caesarian section	0.6	0.2, 1.9	0.35	-	-	-	-	-	-

Notes: Depression, PHQ-9 score ≥ 10; anxiety, GAD-7 score ≥ 10, SES, socio-economic status; IPTp, intermittent preventive treatment in pregnancy

1 Multivariate linear regression using depression as predictor

2 Multivariate linear regression using anxiety as predictor

We also assessed whether mothers with comorbidity of depression and anxiety (n = 76) differed from depressed-only (n = 132) or anxious-only (n = 27) or healthy mothers with regard to adverse birth outcomes, and did not find any significant differences.

## Discussion

The results of this study suggest that, in our sample of low-obstetric risk women in sub-Saharan Africa, depression and anxiety during the 3^rd^ trimester per se are not predictive of poor birth outcomes. Although maternal depression and anxiety have been considered to substantially contribute to PTB, LBW and consecutive neonatal morbidity in low- and middle-income countries (LMICs) [10], we cannot confirm this association for otherwise healthy African pregnant women. 

In our sample, maternal depression and anxiety symptoms were quite common while LBW and PTB were rare. Previous studies found that the prevalences of LBW and PTB were between 9 and 17% in the two countries [30,31]. Several reasons likely account for this discrepancy: To control for confounding we restricted our sample to pregnant women without severe pregnancy complications which are known to be associated with poor birth outcomes [32]. The low prevalence of poor birth outcomes may suggest a strong confounding effect of pregnancy complications when examining the association between maternal mental health and birth outcomes. Also, women in Côte d’Ivoire who dropped out of the study due to political instability (n=311) scored higher on mental symptom scales and lower on SES, which may have introduced selection bias towards less severe exposure and, potentially, outcomes. 

CMD during pregnancy have been evaluated in many settings, mainly focusing on depression and anxiety. Reports from high and low-income countries linking maternal CMD to perinatal outcomes showed conflicting results. A study in pregnant women from U.S. minority groups (n=712) found the adjusted odds ratio (OR) for delivering a LBW infant was 3.97 (95% confidence interval (CI) 3.80-4.15) and for PTB was 3.39 (95% CI 3.24-3.56) in adult depressed women as compared to non-depressed women [33]. This association was not present among adolescents. This study excluded pregnant women who had a history of non-obstetric, chronic disease (e.g. insulin-dependent diabetes mellitus or systemic lupus erythematosus), drug or alcohol abuse, or of psychiatric illness before pregnancy, but did not exclude those with pregnancy complications. Another study among mainly African-American women (n=1399) in Baltimore, Maryland, U.S., found maternal depressive symptoms independently associated with spontaneous PTB (OR 1.96; 95% CI 1.04-3.72) [34]. The same research group reported specific pregnancy-related anxiety to be also linked to an increased risk of spontaneous PTB in their sample, while measures of general anxiety symptoms were not applied [35]. In this study first and second-trimester bleeding was the only pregnancy complication adjusted for in the logistic regression model. On the other hand, studies in the U.S. [13,36], Sweden [37], Norway [38,39], and Ethiopia [40] did not find significant associations between maternal mental health and birth outcomes. 

Data from LMICs including Pakistan [12], India [11], Brazil [41], and Bangladesh [15] found associations between depressive and/or anxiety symptoms with LBW and/or PTB, but none of these studies considered the impact of pregnancy complications, and their samples did not exclude women of high obstetric risk. 

Overall, it appears that studies which controlled for pregnancy complications did not find a strong influence of maternal depression and anxiety on LBW and PTB. A systematic review on correlates of anxiety symptoms during pregnancy and the association with perinatal outcome reported that there is no evidence of a relationship between general anxiety symptoms and birth outcomes (PTB, LBW, 5 minutes Apgar score) [42]. Yet, a recent systematic review on the association between depression during pregnancy and PTB, LBW and intrauterine growth restriction found that antepartum depression was associated with LBW and PTB. The risk of LBW associated with antenatal depression was larger in LMICs (relative risk (RR): 2.05; 95% CI 1.43-2.93) compared to the United States (RR: 1.10; 95% CI 1.01-1.21) or Europe (RR: 1.16; 95% CI 0.92-1.47) [10] where effect estimates were only marginally or not significant. Moreover, the pooled effect estimates should be interpreted with caution because the differences across study populations, study designs, sample sizes, confounders, depression and/or anxiety definitions and instruments, and the timing of assessment complicated the comparison of the studies. 

Considering the available data, the heterogeneity of findings is striking. We consider it likely that the literature is confounded by the detrimental effects of pregnancy complications, which have not been accounted for as distinct risk factors for PTB, LBW and other indicators of neonatal ill-health in most studies. 

From an etiological perspective, pregnancy complications can evoke mood disturbances and impair maternal mental health [43]. On the other hand, poor maternal mental health has been found to impact on pregnancy courses through its promoting influence on risk factors for adverse birth outcomes, like unhealthy behavior and poor nutrition [7,44], hypertension and pre-eclampsia [43], uterine infections [9], or lack of prenatal healthcare visits [45]. Altogether, biological pathways of how maternal antepartum depression and/or anxiety could affect birth outcomes are not well understood, and the role of pregnancy complications as confounder, mediator or effect modifier has never been extensively studied. 

Almost none of the studies in LMICs tried to separate the influence of mental disorders per se from pregnancy complications, which may explain the observed larger effects in these countries. To evaluate direct effects of maternal CMD on birth outcomes, we limited the potential effect of pregnancy complications by restricting the study population to physically healthy pregnant women. We did not find significant associations between depression and anxiety during the 3rd trimester of pregnancy and poor birth outcomes.

Consistent with previous data [12,15,40], we found low maternal SES to be negatively associated with child’s weight. Children whose mothers belonged to the top 30% SES group were 200g heavier at birth than children whose mothers belong to the lowest 30% SES group, adjusting for other factors. Although not necessarily associated with impaired health in a specific child, these differences in birth weight are substantial from a population perspective. 

This study has limitations: First, we measured maternal depression and anxiety in the 3^rd^ trimester of pregnancy. Yet, pre-existing mental disorders that may affect fetal growth at earlier gestational stages were not captured by the nature of our study design [46]. Second, the anxiety measure applied is not specific for pregnancy-related anxiety but rather for general anxiety symptoms. Some researchers suggest that pregnancy- and child-directed concerns and worries could be more closely associated with perinatal outcomes than symptoms of general anxiety [47]. Also, the screening instruments used to assess CMD are imperfect and subject to information bias. Third, 311 mothers in Côte d’Ivoire dropping out due to political instability, may have led to selection bias. Forth, by design the results are not generalizable to women with high obstetric risk. Fifth, a lack of statistical power could explain negative results.

Despite these limitations, this study is important because it fills the data-gap on antepartum CMD and birth outcomes in resource-poor settings and in sub-Saharan Africa in particular [48]. To our knowledge, only one study on antepartum CMD and adverse birth outcome (LBW) in sub-Saharan Africa was conducted in Ethiopia [40]. Like almost all data from LMICs, this study did not control for the potential effect of pregnancy complications. Since antepartum CMD are highly prevalent in African pregnant women and pregnancy complications may have particularly severe consequences for the neonate, more studies that carefully control for potential confounders including pregnancy complications are needed to understand the effect of antepartum CMD on the health of neonates in these populations. 

## Conclusions

Our data suggests that maternal depression and/or anxiety in the 3^rd^ trimester of pregnancy are not independent predictors of adverse birth outcomes, such as LBW, PTB, and low Apgar scores in a low obstetric risk population with good but not unusually exceptional antenatal care. Further studies on mental disorders in pregnant women and their impact on birth outcomes are needed. Notably, the role of pregnancy complications as confounders or effect modifiers should be explored.
